# Effects of meteorology and human‐mobility on UK's air quality during COVID‐19

**DOI:** 10.1002/met.2061

**Published:** 2022-05-06

**Authors:** Cammy Acosta‐Ramírez, Jonathan E. Higham

**Affiliations:** ^1^ Department of Geography and Planning School of Environmental Sciences, University of Liverpool Liverpool UK

**Keywords:** air‐pollution, COVID‐19, DEFRA, Google mobility, meteorology, MIDAS

## Abstract

Efforts to prevent the spread of the coronavirus disease 2019 (COVID‐19) pandemic have had profound positive and negative impacts on social and environmental indicators worldwide. For the first time, a scenario of a partial economic shutdown could be measured, and large tech companies published wide‐coverage mobility reports to quantify the impacts on social change with anonymized location data. During the COVID‐19 pandemic, the UK government has employed some of the strictest lockdown periods in the world, causing an immediate halt to travel and business activities. From these repeated lockdown periods, we have gained a snapshot of life without excessive human‐made pollution; this has allowed us to interrogate the interaction between meteorology and air quality with minimal anthropogenic input. Our findings show a warmer 2020 increased the UK's ozone levels by 9%, while reductions in human‐mobility reduced UK‐wide nitrogen dioxide levels by 25% in 2020, which have remained low during the first months of 2021 despite curtailing/ending of restrictions; and a decrease in particulate matter created by meteorological and human drivers. Regionally, London records the highest NO_2_ and O_3_ changes, −31% and 35%, respectively, linked to mobility reductions and meteorology.

## Graphical abstract

First lockdown observed the highest nitrogen dioxide reduction. Nitrogen dioxide and particulate matter reductions linked to reduced mobility. London records highest nitrogen dioxide and ozone changes.

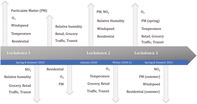

## 1 INTRODUCTION

Numerous studies have shown human activity is the major cause of air pollution (Fu & Chen, 2017; Popescu & Ionel, 2010; Sofia et al., 2020). Continued globalization, consumerism and the intra‐community and international mass transportation of people and goods exacerbate pollution and greenhouse gases (GHGs) production rates (Meng et al., 2016). Despite huge efforts being put in place to reduce these rates, the world continues warming up by 0.3–1.3°C above pre‐industrial levels, and the air quality across the globe has worsened (Sofia et al., 2020; Voosen, 2021).

The relationship between weather and pollution is well documented in the literature. Many studies have established that meteorology plays a significant role in creating, dispersing and transporting pollutants across regions (Cichowicz & Wielgosiński, 2015; Jones et al., 2010). Relative humidity and sunlight are involved in the production of ozone (O_3_) and nitrogen oxides (NO_x_) (Jhun et al., 2015; Kavassalis & Murphy, 2017). Likewise, windspeed has been shown to impact the transport of pollutants; in the United Kingdom, particulate matter shows to have strong correlation with seasonal winds, anticyclonic conditions and long‐range transport from continental Europe (Graham et al., 2020).

Together, human activities and meteorology influence the air chemical composition. But, what if anthropogenic pollution were to stop overnight? How would the atmosphere react? Soon after being declared an international public health emergency (World Health Organization, 2020), public decisions to tackle the spread of coronavirus disease 2019 (COVID‐19) were implemented worldwide. The sudden closing of businesses and raising infections burst health systems, untethered worldwide social and economic structures and for a brief period brought the world to a standstill. From an environmental perspective, these abrupt changes presented an opportunity to investigate natural connections between meteorology and air pollution with minimal anthropogenic input: reduction in traffic, social mobility and industry in contrast with a ‘business as usual’ scenario.

Many COVID‐19 air pollution studies in environmental sciences focussed on (a) changes in GHG emissions and air quality under lockdown (Conticini et al., 2020; Copat et al., 2020; Venter et al., 2020) and (b) the association between pollution levels and meteorology variables with the prevalence of respiratory diseases, including COVID‐19 (Cartenì et al., 2020; Coccia, 2021; Islam et al., 2021; Wu, Jing, et al., 2020).

In a study by Coccia (2021), it was shown low wind speed to be associated with higher concentration of pollutants and indirectly promote the permanence of viral particles and diffusion of coronavirus. In contrast, temperature was found to be inversely correlated with COVID‐19 infections (Cartenì et al., 2020; Islam et al., 2021). A 1°C increase was associated with a 3.08% reduction in new daily cases. Likewise, a 1% increase in relative humidity was linked to a 0.85% reduction in daily new cases (Wu, Jing, et al., 2020).

Despite lockdown measures have been referred as the ‘largest scale experiment ever’ in terms of GHG reduction (Watts & Kommenda, 2020), air pollutants have been linked to the severity of the pandemic. Nitrogen dioxide (NO_2_) and particulate matter with a Sauter mean diameter of 2.5 μm (PM2.5) have been examined due to their role in the COVID‐19 spread and severity (Conticini et al., 2020; Copat et al., 2020; Hendryx & Luo, 2020; Ogen, 2020). An increase of 1 μg/m^3^ in PM2.5 has been to an 8% increase in COVID‐19 death rate (Wu, Nethery, et al., 2020).

Throughout 15 months of pandemic, social restrictions worldwide have been key to control the viral spread (Yechezkel et al., 2021). The unprecedented release of mobility changes by Google (Google Community Mobility Reports, 2021) has revealed how communities responded to the pandemic at a country level and regional level. This release constitutes a significant evidence given the high market share of smartphones held by Google. In the United Kingdom, this involves 48% of smartphone users (~21 million adults, from which users who opted‐in to Location History make up the report. ONS Internet Access, 2020; ONS Population Estimates, 2021; StatCounter, 2021).

As demonstrated by Jordan et al. (2020), there is no sole element determining the severity of the pandemic—or its impact on the environment—but a combination of several factors. The increasing availability of high‐coverage and granularity datasets of mobility, weather and pollution presents us with the opportunity of comparing side by side these spatiotemporal variables and understand their interactions.

In this study, we focus our analysis on the United Kingdom; we use open‐access datasets from government‐funded sources to obtain pollution and weather data from 2020 to mid‐2021. We use meteorological, pollution and mobility data from all 12 regions of the United Kingdom to investigate their relationships. Hourly meteorological data (temperature, relative humidity and wind speed) are sourced from Met. Office, MIDAS Land and Marine Surface Station Data; and hourly pollution datasets (NO_2_, O_3_, PM10 and PM2.5) are sourced from the Department for Environment, Food and Rural Affairs (DEFRA) AURN (Automatic Urban and Rural Networks).

To determine UK‐wide results, we average results from all of the stations, for the UK regional measures (i.e., those based on UK government office regions) we average from stations in each region. We describe the data using two statistical methods (1) *z*‐score values to demonstrate the relationship of the data to the average values and to examine trends within the data, calculated as follows:

$$\text{baseline}\;z_{i}=\frac{x_{i}-x_{\text{baseline}}}{s_{\text{baseline}}},$$

where $\;x_{\text{baseline}}$ is mean of values from baseline period. $\;s_{\text{baseline}}$ is standard deviation of values from baseline period.

(2) Daily percentage change from the ‘baseline’, we derive this ‘baseline’ by averaging daily measurements from 2017 to 2019, that is, and average of 3 years before the impact of the pandemic.

The article is structured as follows: First, we investigate the connections between changes in air pollution and meteorological conditions examining links between weather patterns and sudden changes in air pollution. Second, we examine the changes in air pollution related to social restrictions (i.e., imposed by UK government). Finally, we consider the links between meteorological conditions, air pollution levels and mobility. The overall aim of the article is to untangle the complex relationship between all these factors with the goal of deciphering which factor has been the biggest driver of air‐pollution change during the COVID‐19 pandemic.

## 2 UK‐WIDE METEOROLOGY AND AIR‐QUALITY LEVELS

**FIGURE 1:**
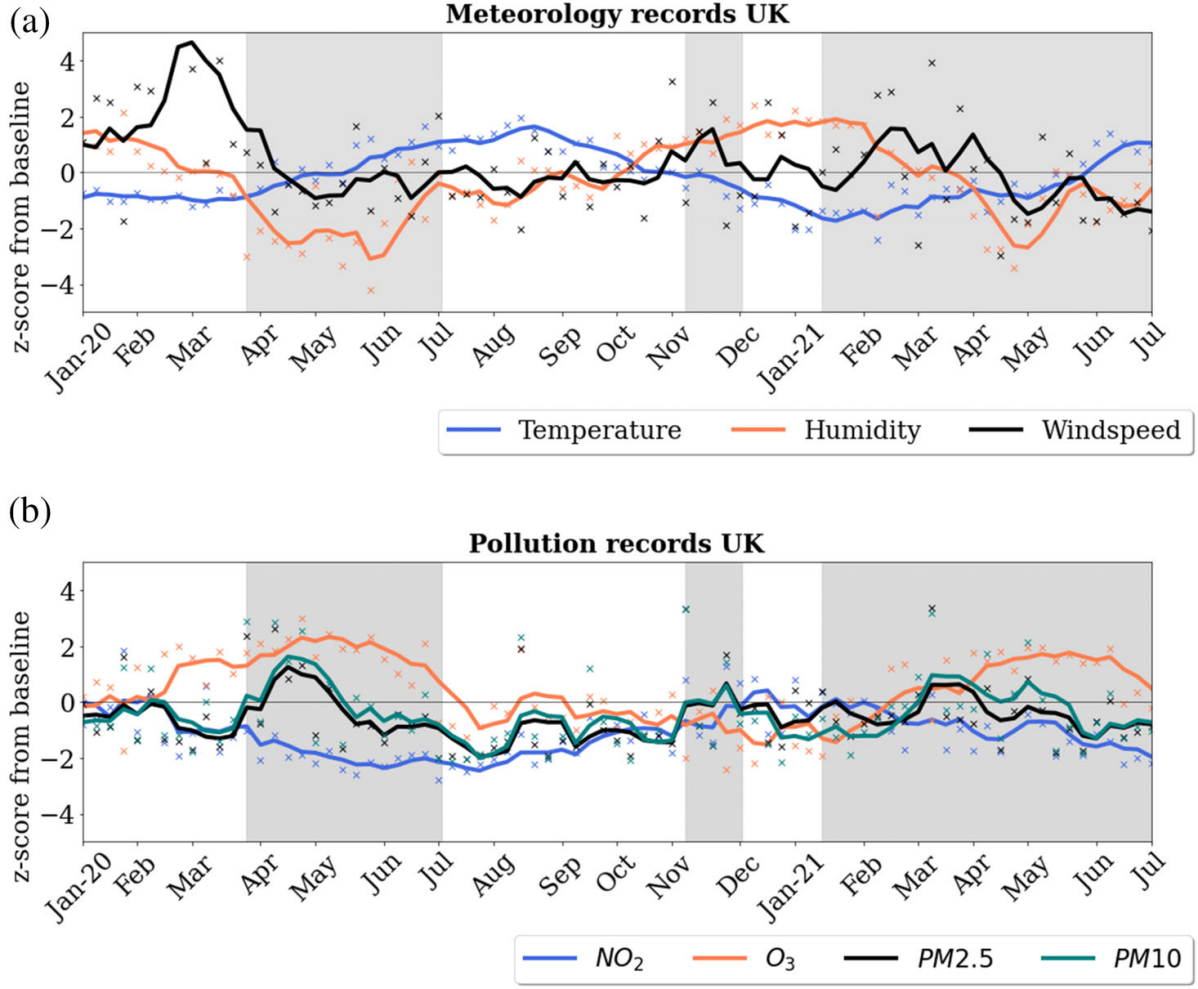
(a) Time series of *z*‐scores from baseline: Temperature, relative humidity and wind speed values. (b) Time series of *z*‐scores: NO_2_, O_3_, PM2.5 and PM10. Successive lockdowns highlighted in grey

**FIGURE 2:**
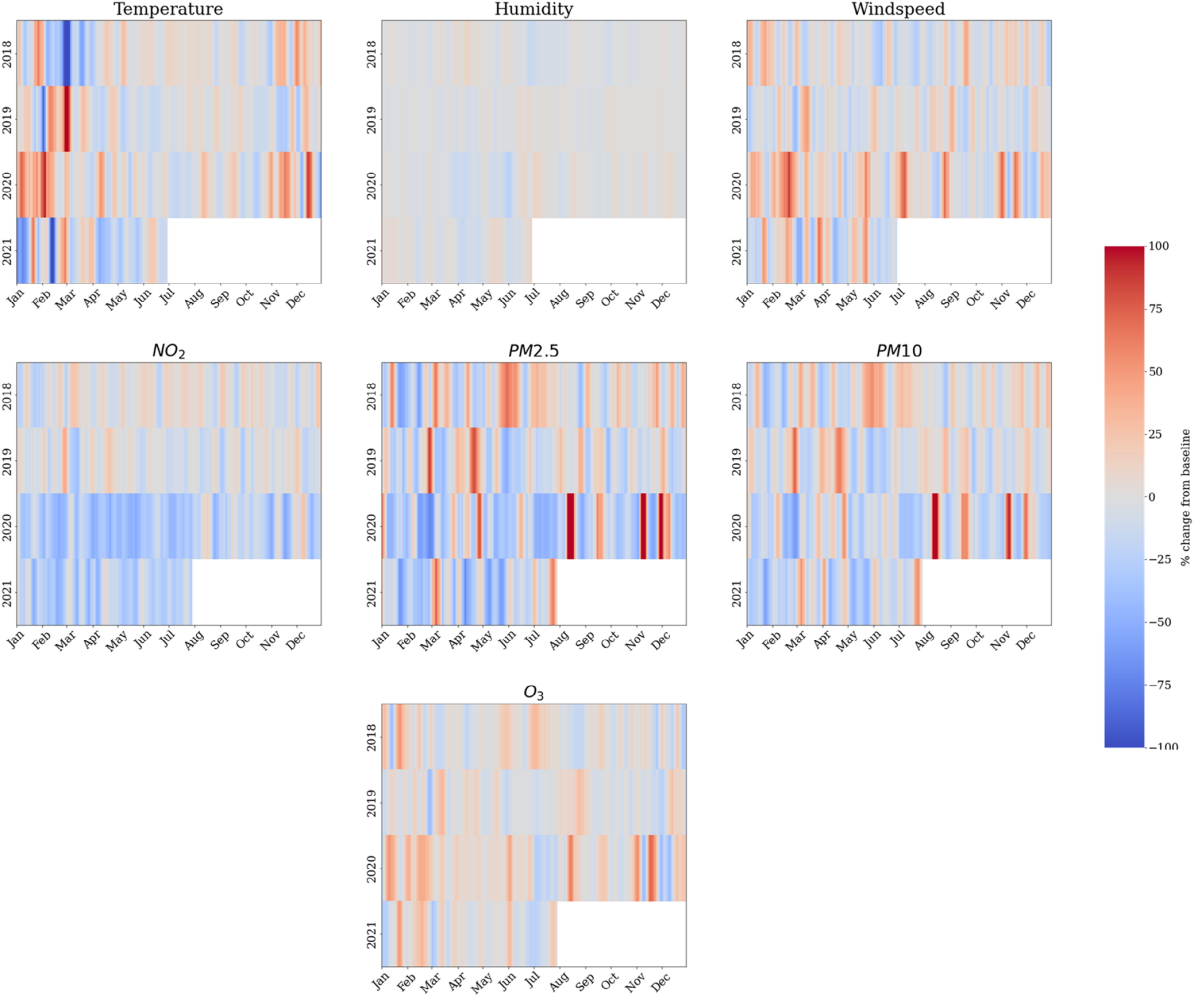
Temporal representation of percentage change in meteorological and pollutant quantities compared with 3 years precoronavirus disease 2019 (pre‐COVID‐19) baseline

In Table 1, we show the year statistics of the meteorological and pollution quantities; these results present the percentage deviations from the baseline levels (determined the daily averages of the previous 3 years 2017–2019), for completeness, we include the raw values. In Figure 1, we present time series *z*‐score values of the meteorological and pollution data, showing the trends of the quantities throughout the COVID‐19 pandemic. The *z*‐scores represent a standardized value of each metric (both meteorological and pollutants) to allow for a better understanding of relative trends and changes, which have occurred as a result of successive lockdowns. In Figure 2, we present the time series of percentage differences (2017–2021) from the baseline of the meteorological quantities. These statistics allow us to compare the fluctuations from the baseline for the entire period 2017–2021.

**TABLE 1:** Recorded values for meteorological conditions and air pollution, a yearly average from daily/hourly records

Variable (unit)	2017	2018	2019	2020	2021^a^
Temperature (°C)	10.19	10.20	10.08	10.34	7.65
% var. from baseline	0.90	−1.98	1.08	9.28	−11.50
Humidity (%)	83.29	81.59	82.44	81.23	80.52
% var. from baseline	1.10	−1.08	−0.03	−1.45	−0.93
Windspeed (kn)	8.86	8.74	8.59	9.39	8.48
% var. from baseline	1.74	−0.24	−1.50	10.77	−0.36
NO_2_ (μg/m^3^)	24.92	23.81	23.20	17.39	18.22
% var. from baseline	2.64	0.43	−3.07	−25.43	−22.20
O_3_ (μg/m^3^)	47.55	50.24	49.26	52.79	54.62
% var. from baseline	−2.56	1.98	0.58	9.16	1.47
PM2.5 (μg/m^3^)	9.70	9.95	9.75	7.85	8.45
% var. from baseline	−1.50	3.56	−2.06	−10.44	−14.72
PM10 (μg/m^3^)	15.50	16.33	16.32	14.32	15.00
% var. from baseline	−3.87	3.43	0.45	−5.95	−10.00

*Note*: The variation from baseline is calculated for each day and averaged yearly.

a Time period January–July.

The data in Table 1 clearly show during successive lockdowns (2020–2021), significant reductions in NO_2_ (−25.4% and −22.2%), PM2.5 (−10.4% and −14.7%) and PM10 (−5.9% and −10.0%), similar statistics have been widely reported in other journal articles (Berman & Ebisu, 2020; Siddiqui et al., 2020). In line with the findings of Higham et al. (2020) in 2020, the data show an increase in O_3_; however, this has reduced in 2021, as shown in Figures 1 and 2, this coincides with a particularly warm start and end to the year 2020 (9.3% increase) and 2021 with a cold first half of the year (11.5% decrease). Table 1 further depicts 2020 as a particularly windy year, with an overall 10.8% increase versus the 2017–2019 baseline, with values close to baseline during 2021. Figure 2 shows this related to a very windy 4 month start to 2020 with ~40% greater than the baseline winds. Overall, PM2.5 and PM10 reduced significantly in 2020 and 2021, while it is extremely likely a portion of the reduction relates to dispersion, it is also very likely reduced human movement will have affected the levels of PM2.5. The reduction in 2021 of particulate matter reached 1.5 standard scores from the baseline; on closer inspection of Figures 1 and 2, it is apparent that the heavy winds reduced the levels of particulate matter initially, after which there are spikes in particulate matter. In 2021, it is likely the lockdowns have had the biggest effect of reductions in PM10 and PM2.5; however, successive spells of short windy events have likely kept particulate matter levels low (Figures 1 and 2).

### 2.1 Regional meteorology and air pollution

**FIGURE 3:**
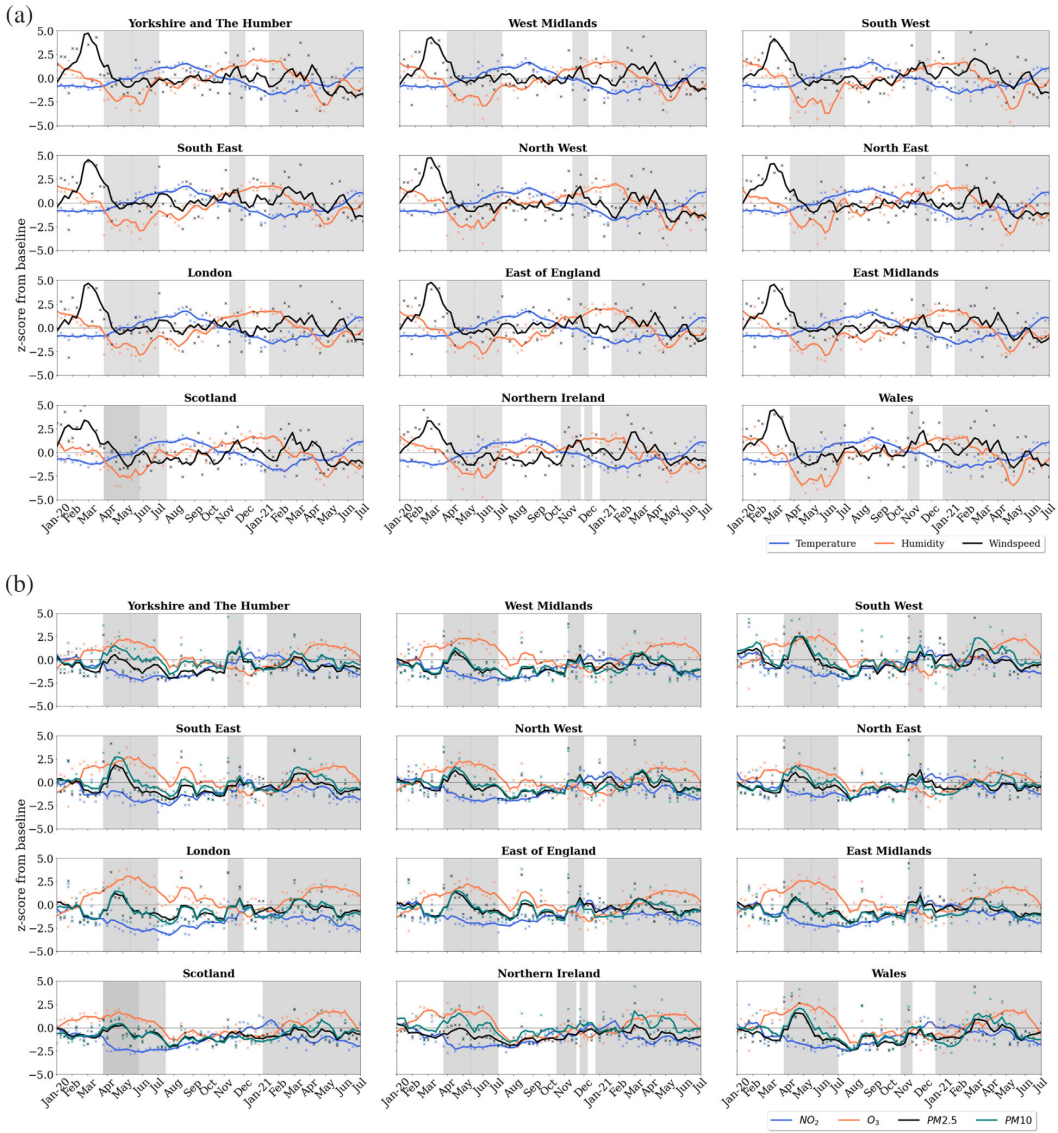
(a) Regional time series of *z*‐score for temperature, relative humidity and wind speed values. (b) Regional time series of *z*‐score for NO_2_, O_3_, PM2.5 and PM10. Successive lockdowns highlighted in grey

**FIGURE 4:**
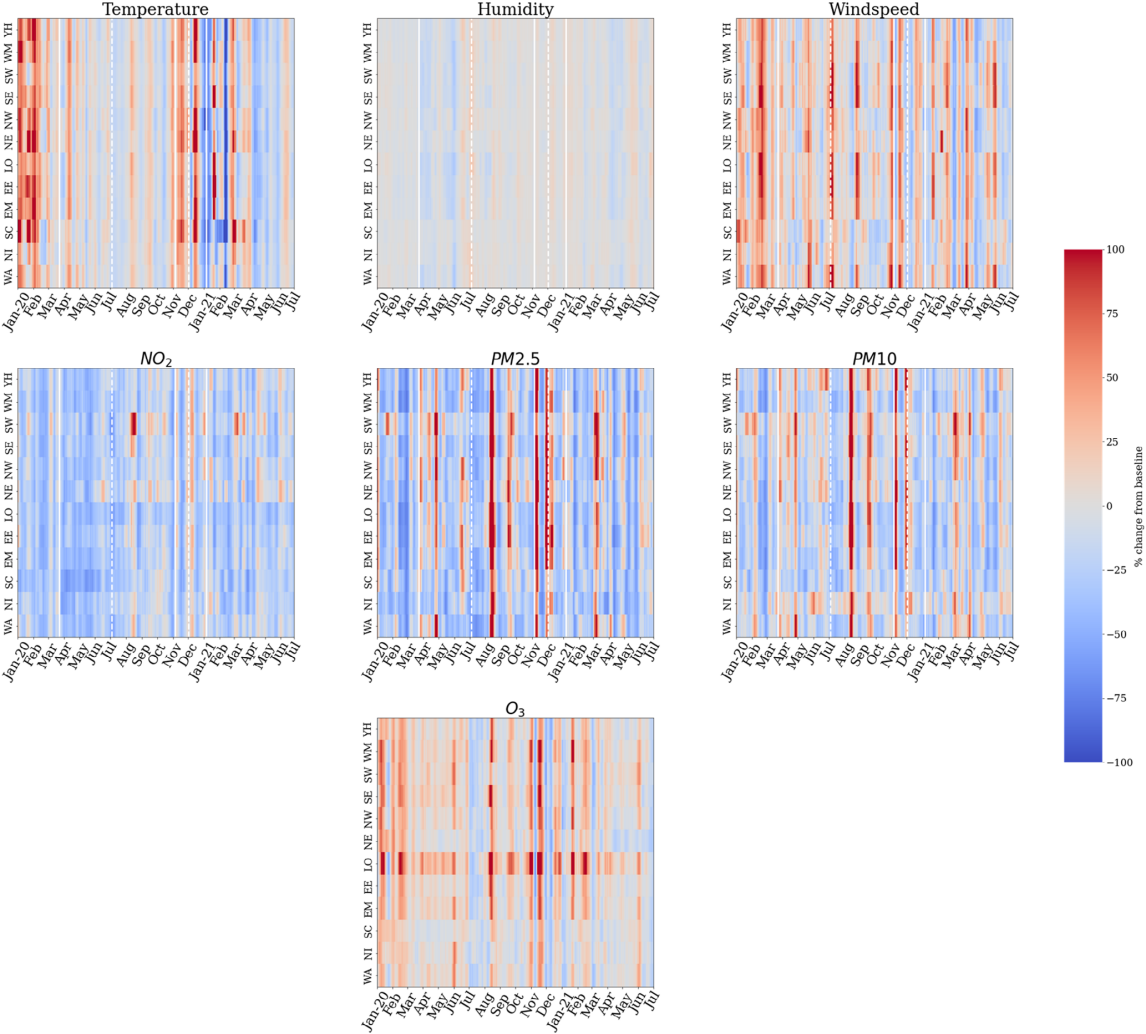
Regional temporal representation of percentage change in meteorological and pollutant quantities (2020–2021). White solid lines denote start of lockdown, white dashed lines denote end of lockdown. Regions presented as: (i) YH, Yorkshire and the Humber, (ii) WM, west midlands, (iii) SW, south west, (iv) SE, south east, (v) NW, north west, (vi) NE, north east, (vii) LO, London, (viii) EE, east of England, (ix) EM, east midlands, (x) SC, Scotland, (xi) Northern Ireland and (xii) Wales

Regional meteorological quantities are shown in Figure 3 for their potential association with pollution changes. Across the regions, the reductions in pollutants (Figures 3 and 4) are linked to the lack of human activity and meteorological conditions. An unseasonably warm start to 2020 is observed in the temporal representation of daily changes (Figure 4), in some regions reaching twice the temperature of the baseline years. Following the UK‐wide findings, the increases in temperature relate to an increase in O_3_. Interestingly, regions such as Scotland and Wales show O_3_ are lower with warm temperatures during the warmest months of 2020; this could be related to cloud cover and an increase in relative humidity, reduction in NO_x_ and reductions in volatile organic compounds (VOCs) as described by Lacour et al. (2006). Higher temperatures continue in the summer of 2020 and decrease during autumn and winter. From spring 2021, periods of warm temperature cause a second resurgence in the O_3_ levels. Following the UK‐wide findings connecting wind speed and particulate matter we find that, across the regions with heavier winds, there were more significant decreases and increases in particulate matter levels. Similarly, short spells of high‐intensity winds in rapid succession have kept the particulate matter levels low.

From both a UK wide and a regional perspective, the warm start and end to 2020 and the reduction in NO_x_ caused peaks in O_3_ production. Additionally, intermittent periods of high and low wind speed promoted the dispersion and deposition, respectively, of particulate matter, with shorter intense windy periods keeping particulate matter levels low in 2021. However, considering that around half of UK concentrations of particulate matter comes from anthropogenic sources (DEFRA, 2021), the patterns observed during the COVID‐19 pandemic are evidently related to the sudden change in human mobility.

## 3 UK GOVERNMENT‐IMPOSED LOCKDOWNS AND MOBILITY

Starting from the 23rd March 2020, the UK government implemented the largest set of restrictions ever known. First thought to be a single set of restrictions, three successive periods of social and economic lockdowns were imposed (each with different restriction levels) and different times in the year. We summarize these dates, based on majority England (largest population below):

1. Lockdown 1—23rd March, 2020 to 4th July, 2020.
2. Lockdown 2—5th November, 2020 to 2nd December, 2020.
3. Lockdown 3—6th January, 2021 to 19th July 2021.

**FIGURE 5:**
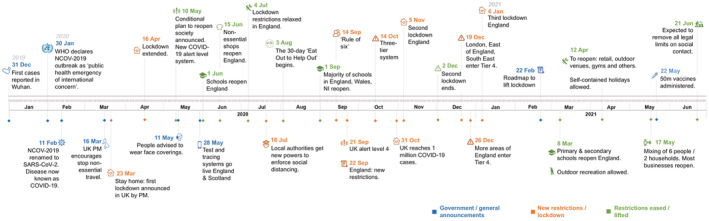
Timeline of significant events during the coronavirus 2019 (COVID‐19) pandemic in the United Kingdom

The timeline of measures is summarized in Figure 5. To quantify these changes in mobility, we use Google Community Mobility Reports (2021). These aggregated, anonymized data show how busy certain type of places are based on the users with location history activated on devices with a Google Account. The categories selected are Retail and Recreation, Grocery and Pharmacy, Transit stations and Residential. The reported baseline is the median value, for the corresponding day of the week during the 5‐week period January 3rd to 6th February, 2020.

Considering that the Transit category in Google measures visits to public transport hubs such as train stations and buses, an additional Traffic mobility measure was included. The time series, sourced from the UK Department of Transport (Domestic transport use by mode: since 1 March 2020), reports a baseline of the equivalent day in the first week of February 2020.

## 4 UK‐WIDE MOBILITY AND AIR QUALITY LEVELS

**FIGURE 6:**
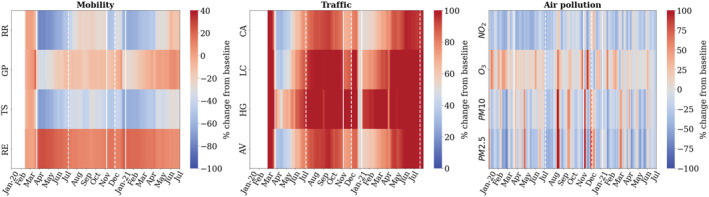
UK wide temporal representation of percentage change in (a) mobility (2020–2021), (b) traffic and (c) pollutant quantities (2020–21). Mobility categories presented as: (i) RR, retail and recreation, (ii) GP, grocery and pharmacy, (iii) TS, transit stations and (iv) RE, residential. Traffic categories presented as: (i) CA, cars, (ii) LC, light commercial vehicles, (iii) HG, heavy goods vehicles, (iv) AV, all motorized vehicles

**FIGURE 7:**
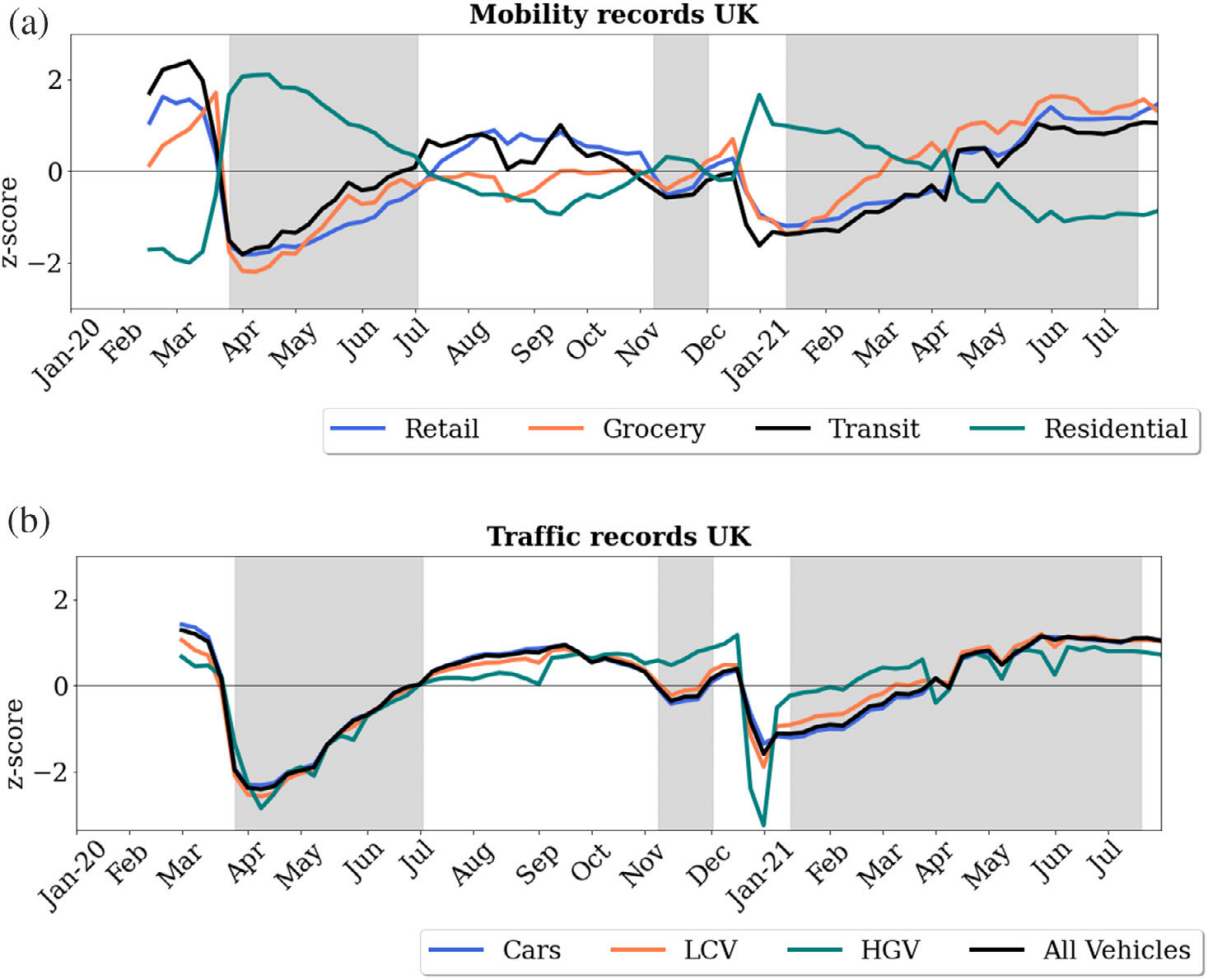
(a) Google mobility standard scores. (b) Traffic in the United Kingdom during coronavirus disease 2019 (COVID‐19) pandemic, standard scores. Traffic presented as: (i) cars, (ii) LCV, light commercial vehicles, (iii) HGV, heavy goods vehicles, (iv) all vehicles

Figure 6 shows a temporal representation of percentage difference between the reported baseline (median value of the corresponding day of the week during January 3rd–6th February, 2020; Google, 2021) of UK‐wide mobility based on retail, grocery, transit and residential, traffic and air pollutant levels. In Figure 7, we present the *z*‐score index of mobility and traffic changes depicting the trends relating to successive lockdowns and changes in human activity. Across the United Kingdom, the sudden changes in mobility caused by subsequent lockdowns had a significant impact on air quality. The most significant changes are observed in Transit stations and Traffic for all vehicles, with reductions over 70% versus baseline during the first weeks of lockdown. Similarly, Retail and Recreation showed significant reductions of over 60% against the baseline.

During the first lockdown, NO_2_ values were on average 36% less than the baseline. This downward trend in NO_2_ reduction continued after lifting restrictions but started rising slowly from July 2020. Concentrations of NO_2_ from then on began to steadily increase proportional to the levels of Traffic and Transit but also inversely proportional to Residential. The NO_2_ levels peaked back to the baseline levels in December 2020 (Christmas relaxed period). The third lockdown observed similar environmental changes as those observed in the first lockdown gradual decrease in NO_2_. From April 2021, mobility increased strongly to reach values close to baseline; however, NO_2_ remained low. In contrast with the first lockdown records, a −17% average reduction in NO_2_ was observed during the second lockdown and −22% during the third, which is related to meteorology conditions and the increases in Traffic shown in Figure 7. Whilst there were changes in particulate matter, the magnitude of the observed decreases was not of the order of that of nitrogen dioxide. The observed fluctuations, an increase during the first weeks of lockdown and a gradual reduction to values close to the baseline throughout the 15 months of restrictions, could reflect an annual cyclicity at the beginning of the summer and its interaction with meteorology variables.

Notably within the mobility data and pollution, we also see several spikes; these spikes correspond to notable events during the pandemic. In the week before the nationwide lockdown, reflecting the ‘panic‐buying’ amidst the pandemic uncertainty causing a sudden jump in NO_2_ levels by ~10%. Similarly, high peaks were observed in December, related to Christmas holiday shopping, and visits kept rising in 2021, reaching levels the baseline and NO_2_/particulate matter levels and almost at baseline concentrations.

## 5 REGIONAL MOBILITY AND AIR‐QUALITY LEVELS

**FIGURE 8:**
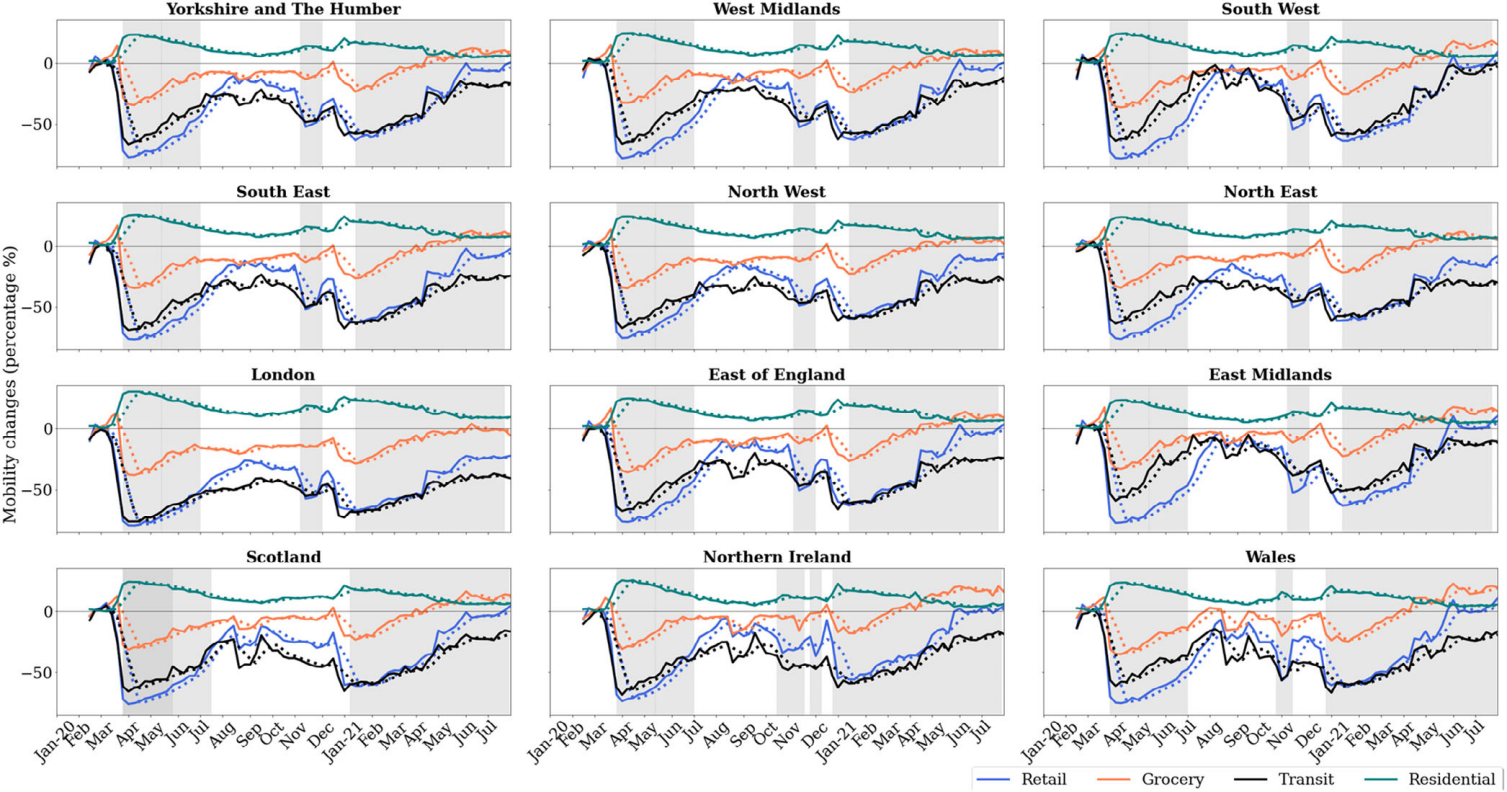
Time series of mobility. Percentage changes from baseline: Retail, Grocery, Transit stations and Residential categories. Successive lockdowns highlighted in grey

Mobility records from Google are reported from mid‐February and show a massive drop in visits to Transit stations and Retail from the beginning of lockdown for all the UK regions (Figure 8). Both categories showed similar trends throughout lockdown. As the weeks after March 23rd progressed, visits increased gradually to reach a peak between July and September. From mid‐August to September, slight reductions in Transit and Retail were recorded in some regions, which could reflect the public reaction to cases increasing after restrictions were lifted in July. The most significant drop, observed in Scotland for both Transit and Retail, might be partially related to a local lockdown in Aberdeen and the government's response to the crisis, considering retail as a risk factor (BBC News, 2020a). Similar restrictions were introduced in Northern Ireland during August (BBC News, 2020b).

Simultaneously, an increase in Retail was observed in most regions during August, related to ‘Eat Out to Help Out’ scheme (a scheme devised by the UK chancellor to promote business in hospitality venues). From mid‐September, visits decreased to a significant drop during the second lockdown, to subsequently peak in mid‐November in Wales and the start of December in England. It is noteworthy that during this period, regional and national variations reflect the different restrictions in England, Wales and Northern Ireland, highlighting the critical importance of government decisions on mass social mobility. For all regions, Transit and Retail reduced drastically in late‐December during Christmas holidays and continued increasing slowly until a sudden peak in the between the 12th and 18th of April in England and Wales, when retail and close contact businesses reopened (Figure 5 **)**.

**FIGURE 9:**
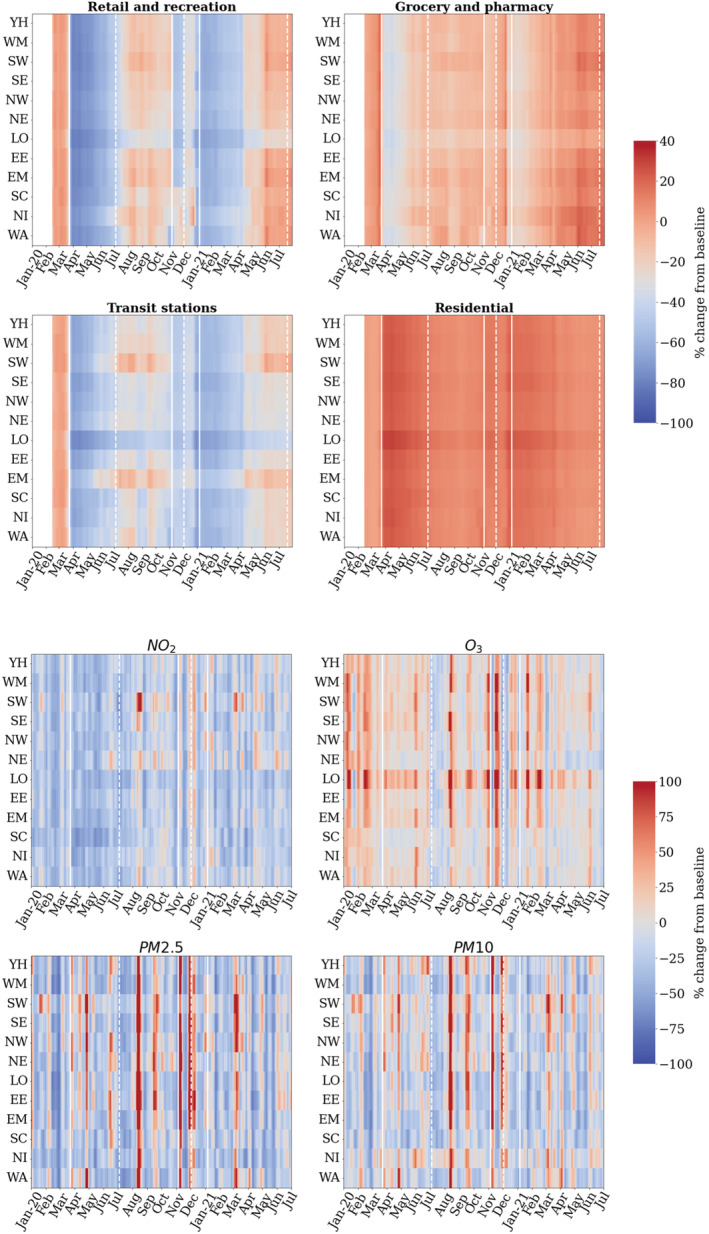
Regional temporal representation of percentage change in mobility (a) and pollutant records (b)during 2020–2021. White solid lines denote start of lockdown, white dashed lines denote end of lockdown. Regions presented as: (i) YH‐Yorkshire and the Humber, (ii) WM, west midlands, (iii) SW, south west, (iv) SE, south east, (v) NW, north west, (vi) NE, north east, (vii) LO, London, (viii) EE, east of England, (ix) EM, east midlands, (x) SC, Scotland, (xi) Northern Ireland and (xii) Wales

Residential category percentage changes range between 0% and 30% on average (Figure 8). The less variation observed in this category (compared to Retail, Grocery and Transit) derives from the nature of measurements, which indicate a change in *duration* instead of visits. Because duration has a limit of 24 h in a day, and people already spend a significant part of the day at places of residence, the largest possible change might be +50% or less during weekends (Google, Community Mobility Reports Help, 2021b). The most significant change at the beginning of lockdown was observed in London, with an increase of 30% in the time spent at home, followed by South West and Scotland (Figure 9). The majority of regions increased their residential duration by 23%–25% on average. As the months progressed in 2020, the time spent at residential places was gradually reduced, reaching the lowest percentage of change in September. Later, residential duration increased gradually, to an average of 15% of increase during second lockdown in November in England and late‐November and beginning of December in Wales. Northern Ireland also showed an increase during strict lockdown restrictions from mid‐October. During Christmas holidays, time spent at home increased by 20% in all regions and kept decreasing during 2021 to reach values close to baseline from June onwards.

As observed in Figure 3 and the temporal distribution in Figure 8 (bottom), NO_2_ levels started to drop at different dates between regions, in Scotland and Northern Ireland, NO_2_ pollution starts reducing right after the beginning of the first lockdown, while the majority of regions shows NO_2_ values decreasing consistently from mid‐February before the start of lockdown. These decreasing values are related to higher wind speed during February (Figure 3). However, regions show mixed results despite a very similar increase in wind speed. London, the most densely populated region, had the most pronounced NO_2_ reduction, highlighting the contribution of anthropogenic activities following the confirmation of COVID‐19 cases in the country. This contribution could have been more significant than wind speed in NO_2_ levels during March. As the wind speed returned to ‘normal’ at the start of April, an increase of +15% in NO_2_ levels was registered but as noted in Figure 3, it was promptly dissipated as less human mobility meant less NO_2_ emissions.

While mobility started recovering slowly, the reduction in NO_2_ showed consistency during the year and amongst regions, with a gradual increase from mid‐July after restrictions were lifted. This gradual increase peaked at the beginning of December in English regions, associated with winter meteorological conditions, restrictions lifting and heavy goods vehicles traffic (Figure 7), and started decreasing considerably once more after the third lockdown was imposed in January 2021. During the same period (December–January), windspeed role is also highlighted in Scotland, Northern Ireland, North East and Yorkshire, where high concentrations of NO_2_ were recorded despite reductions in mobility. This could be related to a heavier use of combustion power plants for wintertime home heating (supported by the Residential category increase) and less sunlight. As the windspeed rose in these regions (Figure 3), NO_2_ levels reduced and continued decreasing gradually during the third lockdown.

Ozone trends contrast those of NO_2_ as observed in the temporal representation of Figure 8. For all regions, increased values were observed during the first lockdown. After lifting restrictions, changes in O_3_ versus the 2017–2019 baseline were less prominent, with values near and below the baseline mean for most regions, with the exception of South East, East of England and London and East Midlands, which exhibited an O_3_ increase during the August and September. Values increased once more during the second lockdown and at the beginning of the third lockdown, exceeding the baseline levels by 40% in London. As the vast majority of O_3_ is formed in the air from reactions with other pollutants (i.e., VOCs and NO_x_), the contrasting trend with NO_2_ is expected, as well, warm and sunny days with lower wind speed promote its production, as supported by the increases in the summer months presented in Figures 3 and 8.

On the other hand, PM10 and PM2.5 show similar patterns between each other, with PM10 showing slightly higher values than PM2.5, particularly for the regions Yorkshire, South East, South West and Northern Ireland (Figure 8). For both pollutants, the first 6 weeks of lockdown recorded a significant rise versus baseline mean, right after the extreme wind speed events recorded in mid‐February to mid‐March (Figure 3), which shows the relationship between wind speed events and the concentration and dispersion of dust. From the end of April, a decreasing trend was recorded until August, when a significant increase in PM10, PM2.5 and O_3_ values registered in most regions. This peak matches the increase in Retail during August, suggesting a potential connection between them. Slight increases and reductions continued throughout the year. During the second lockdown in the first half of November, a particulate matter peak higher than the baseline was recorded in English regions, right after a wind speed peak at the end of October and the Bonfire Night (5th November) in the first week of November. After this event, particulate matter pollution decreased during December and increased slightly during the end of 2020 and start of 2021, likely related to increased heating and fireplaces in the winter. Records from the third lockdown show a slight increase for both PM2.5 and PM10 from February 2021, particularly during March the increase was most significant in South West, South East and Northern Ireland, which could be a consequence of increased wind speed in the prior weeks as displayed in Figure 3.

## 6 DISCUSSION AND CONCLUSIONS

While lockdown restrictions have been in place for over 15 months in the United Kingdom, variations between regions and the gradual reopening of the economy have been reflected in the social mobility changes per region and their pollution trends. At the beginning of the lockdown, most pollution studies hypothesized about reductions in mobility benefiting the environment by reductions in GHGs emissions. As months passed by, a mixed scenario between gas pollutants and particulate matter was observed globally, and interactions between them were further described (Huang et al., 2021).

In the United Kingdom, the drastic changes in mobility at the start of the first lockdown were reflected in NO_2_ reductions across the regions. Studies of reduction in total traffic reported a decrease of around 32% in total traffic (Hicks et al., 2021), and the Department of Transport reports an overall 22.5% reduction in traffic during the 15 months of social restrictions in the United Kingdom. Traffic reduction has been shown to correlate with NO_2_ declines as it accounts for 49% of NO_2_ emissions in the United Kingdom (DEFRA, 2004; Forster et al., 2020). Here, Transit stations and Traffic data show an indirect and direct approach to evaluate NO_2_ trends in relationship with transport. During the first lockdown the depletion in NO_2_ is observed in conjunction with Traffic and Transit stations changes. However, while mobility recovers to baseline levels, NO_2_ continues depleted with a slow increase. While the reduction in NO_2_ continued to be significant throughout the second and third lockdowns, it did not reach the levels of depletion of the first lockdown. Furthermore, for a short period of time, lockdown traffic increased alongside NO_2_ after the second lockdown. The role of meteorology is outlined in this event, since previous increases in Traffic did not result in NO_2_ accumulation, until temperature dropped towards the end of 2020 and periods of low wind speed were recorded, necessary conditions for winter NO_2_ episodes (DEFRA, 2004).

In contrast with NO_2_, O_3_ shows an upward trend from the beginning of lockdown, an inverse relationship with NO_2_ values. As O_3_ is formed from VOCs interacting with NO_x_, changes in NO_x_ at ground level will modify the O_3_ production. NO_x_ also acts as a quencher of O_3_ through NO_x_ titration (Jhun et al., 2015). The observed increase in O_3_ in all regions of UK can be related to a lower O_3_ titration by NO_x_, as well, to the gradual increase in temperature. Other works reported increases in O_3_ during lockdown periods (Higham et al., 2020; Sicard et al., 2020). Another factor influencing O_3_ changes is relative humidity. According to Kavassalis and Murphy (2017), when relative humidity is high, the opening of trees stomata removes O_3_ by dry deposition. This relates to the inverse relationship between O_3_ and relative humidity observed in all regions. The increase in O_3_ and NO_2_ reduction are more evident for London, region in which mobility (Transit stations) fell by 70% and continued with a 50% reduction throughout 2020. As one of the most populated regions with a social influx from different areas of the country that stopped during the pandemic, these pollution changes illustrate the contribution of human‐made sources to regional air quality in an urbanized environment.

The trends we find in particulate matter concentration can also be explained by Hicks et al. (2021) who reported that in the United Kingdom, the average speed in road transport increased by 15% during lockdown due to having less traffic. Particulate matter generated from moving vehicles (non‐exhaust particulate matter) varies widely in its physical and chemical composition, and the reduction in traffic during lockdown restrictions might have caused, indirectly, an increase in non‐exhaust particulate matter. Also, the use of fireplaces and stoves, sources of primary particulate matter and considered the largest single source in the United Kingdom (DEFRA, 2019), could emit particulate matter that is further transported between regions or resuspended (Jones et al., 2010). This is supported by peaks in both PM10 and PM2.5 immediately after peaks in wind speed.

In conclusion, COVID‐19 and the related imposed social restrictions have given us an insight into what air pollution levels might be like in the United Kingdom if we manage to reduce out pollution outputs significantly over the coming years. It is a well‐known fact that meteorology and air pollution are closely linked, and the atmosphere is more than capable of removing its own pollution. However, in this study, we find, those pollutants which can have the biggest impact on human health (i.e., particulate matter and NO_2_) are only reduced by reducing human activity in combination with meteorological conditions that interact with the resultant pollution changes to promote a ‘clean air’. As well, we found that reductions in mobility show regional features with a proportionality to the pollutant changes, as observed for London; and that similar pollution events would have a different duration depending on the regional meteorology, as observed in the increases in NO_2_ at the end of 2020. Ozone created by the atmosphere during 2020–2021 has been found to have increased. Although these levels are relatively low, their concentrations and their related chemical reactions are likely to have little impact in comparison with human‐made pollutants. Returning to our earlier question, if anthropogenic pollution were to stop overnight, how would the atmosphere react? From our findings, while the pollution short term effects of reduced mobility are dramatic, the meteorology conditions hold predominance over human mobility to determine how clean the air is at a regional level. These findings demonstrate the importance of considering regional meteorology and seasonal changes while developing strategies to improve air quality: these relationships are complex, and there is much more work to be done to gain a detailed understanding.

## AUTHOR CONTRIBUTIONS

**Cammy Acosta‐Ramírez:** Conceptualization (equal); data curation (equal); formal analysis (equal). **Jonathan E. Higham:** Conceptualization (equal); data curation (equal); formal analysis (equal); supervision (lead).

## References

[met2061-bib-0001] BBC News . (2020a) Coronavirus: Aberdeen cluster forces pubs to close and more jobs at risk. Available from: https://www.bbc.co.uk/news/uk-53667954 [Accessed 4th August 2021].

[met2061-bib-0002] BBC News . (2020b) Coronavirus: Covid‐19 restrictions 'not aboutscaremongering. Available from: https://www.bbc.co.uk/news/uk-northern-ireland-53860941 [Accessed 4th August 2021].

[met2061-bib-0003] Berman, J.D. & Ebisu, K. (2020) Changes in U.S. air pollution during the COVID‐19 pandemic. Science of the Total Environment, 739, 139864. 10.1016/j.scitotenv.2020.139864 32512381 PMC7442629

[met2061-bib-0004] Cartenì, A. , Di Francesco, L. & Martino, M. (2020) How mobility habits influenced the spread of the COVID‐19 pandemic: results from the Italian case study. Science of the Total Environment, 741, 140489. 10.1016/j.scitotenv.2020.140489 32599395 PMC7313484

[met2061-bib-0005] Cichowicz, R. & Wielgosiński, G. (2015) Effect of meteorological conditions and building location on CO_2_ concentration in the university campus. Ecological Chemistry and Engineering, 22, 513–525. 10.1515/eces-2015-0030

[met2061-bib-0006] Coccia, M. (2021) How do low wind speeds and high levels of air pollution support the spread of COVID‐19? Atmospheric Pollution Research, 12, 437–445. 10.1016/j.apr.2020.10.002 33046960 PMC7541047

[met2061-bib-0007] Conticini, E. , Frediani, B. & Caro, D. (2020) Can atmospheric pollution be considered a co‐factor in extremely high level of SARS‐CoV‐2 lethality in Northern Italy? Environmental Pollution, 261, 114465. 10.1016/j.envpol.2020.114465 32268945 PMC7128509

[met2061-bib-0008] Copat, C. , Cristaldi, A. , Fiore, M. , Grasso, A. , Zuccarello, P. , Signorelli, S.S. et al. (2020) The role of air pollution (PM and NO_2_) in COVID‐19 spread and lethality: a systematic review. Environmental Research, 191, 110129. 10.1016/j.envres.2020.110129 32853663 PMC7444490

[met2061-bib-0009] DEFRA . (2004) Nitrogen dioxide in the United Kingdom. London: Air Quality Expert Group, p. 3.

[met2061-bib-0010] DEFRA . (2019) Clean air strategy. London: Department for Environment, Food and Rural Affairs, p. 3.

[met2061-bib-0011] DEFRA . (2021) Emissions of air pollutants in the UK—Particulate matter (PM10 and PM2.5). Available from: https://www.gov.uk/government/statistics/emissions-of-air-pollutants/emissions-of-air-pollutants-in-the-uk-particulate-matter-pm10-and-pm25. [Accessed 4th August 2021].

[met2061-bib-0012] Forster, p.M. , Forster, H.I. , Evans, M.J. , Gidden, M.J. , Jones, C.D. , Keller, C.A. et al. (2020) Current and future global climate impacts resulting from COVID‐19. Nature Climate Change, 10, 913–919. 10.1038/s41558-020-0883-0 PMC7427494 32845944

[met2061-bib-0013] Fu, H. & Chen, J. (2017) Formation, features and controlling strategies of severe haze‐fog pollutions in China. Science of the Total Environment, 578, 121–138. 10.1016/j.scitotenv.2016.10.201 27836344

[met2061-bib-0014] Google . (2021a) Community mobility reports. Available from: https://www.google.com/covid19/mobility/ [Accessed 18th June 2021].

[met2061-bib-0015] Google . (2021b) Community mobility reports help. Available from: https://support.google.com/covid19-mobility/answer/9824897?hl=en&ref_topic=9822927 [Accessed 18th June 2021].

[met2061-bib-0016] Graham, A.M. , Pringle, K.J. , Arnold, S.R. , Pope, R.J. , Vieno, M. , Butt, E.W. et al. (2020) Impact of weather types on UK ambient particulate matter concentrations. Atmospheric Environment: X, 5, 100061. 10.1016/j.aeaoa.2019.100061

[met2061-bib-0017] Hendryx, M. & Luo, J. (2020) COVID‐19 prevalence and fatality rates in association with air pollution emission concentrations and emission sources. Environmental Pollution, 265, 115126. 10.1016/j.envpol.2020.115126 32806422 PMC7320861

[met2061-bib-0018] Hicks, W. , Beevers, S. , Tremper, A.H. , Stewart, G. , Priestman, M. , Kelly, F.J. et al. (2021) Quantification of non‐exhaust particulate matter traffic emissions and the impact of COVID‐19 lockdown at London Marylebone road. Atmosphere, 12, 190. 10.3390/atmos12020190

[met2061-bib-0019] Higham, J.E. , Ramírez, C.A. , Green, M.A. & Morse, A.P. (2020) UK COVID‐19 lockdown: 100 days of air pollution reduction? Air Quality, Atmosphere and Health, 14, 325–332. 10.1007/s11869-020-00937-0 PMC7485429 32952739

[met2061-bib-0020] Huang, X. , Ding, A. , Gao, J. , Zheng, B. , Zhou, D. , Qi, X. et al. (2021) Enhanced secondary pollution offset reduction of primary emissions during COVID‐19 lockdown in China. National Science Review, 8. 10.1093/nsr/nwaa137 PMC7337733 34676092

[met2061-bib-0021] Islam, N. , Bukhari, Q. , Jameel, Y. , Shabnam, S. , Erzurumluoglu, A.M. , Siddique, M.A. et al. (2021) COVID‐19 and climatic factors: a global analysis. Environmental Research, 193, 110355. 10.1016/j.envres.2020.110355 33127399 PMC7591297

[met2061-bib-0022] Jhun, I. , Coull, B.A. , Zanobetti, A. & Koutrakis, P. (2015) The impact of nitrogen oxides concentration decreases on ozone trends in the USA. Air Quality, Atmosphere and Health, 8, 283–292. 10.1007/s11869-014-0279-2 PMC4988408 27547271

[met2061-bib-0023] Jones, A.M. , Harrison, R.M. & Baker, J. (2010) The wind speed dependence of the concentrations of airborne particulate matter and NOx. Atmospheric Environment, 44, 1682–1690. 10.1016/j.atmosenv.2010.01.007

[met2061-bib-0024] Jordan, R.E. , Adab, P. & Cheng, K.K. (2020) Covid‐19: risk factors for severe disease and death. BMJ, 368. 10.1136/bmj.m1198 32217618

[met2061-bib-0025] Kavassalis, S.C. & Murphy, J.G. (2017) Understanding ozone‐meteorology correlations: a role for dry deposition. Geophysical Research Letters, 44, 2922–2931. 10.1002/2016gl071791

[met2061-bib-0026] Lacour, S.A. , de Monte, M. , Diot, P. , Brocca, J. , Veron, N. , Colin, P. et al. (2006) Relationship between ozone and temperature during the 2003 heat wave in France: consequences for health data analysis. BMC Public Health, 6. 10.1186/1471-2458-6-261 PMC1635711 17054785

[met2061-bib-0027] Meng, J. , Liu, J. , Xu, Y. , Guan, D. , Liu, Z. , Huang, Y. et al. (2016) Globalization and pollution: tele‐connecting local primary PM 2.5 emissions to global consumption. Proceedings of Royal Society A, 472, 20160380. 10.1098/rspa.2016.0380 PMC5134305 27956874

[met2061-bib-0028] Ogen, Y. (2020) Assessing nitrogen dioxide (NO2) levels as a contributing factor to coronavirus (COVID‐19) fatality. Science of the Total Environment, 726, 138605. 10.1016/j.scitotenv.2020.138605 32302812 PMC7151460

[met2061-bib-0029] ONS . (2021) Dataset: estimates of the population for the UK, England and Wales, Scotland and Northern Ireland. Office for National Statistics Available from: https://www.ons.gov.uk/peoplepopulationandcommunity/populationandmigration/populationestimates/datasets/populationestimatesforukenglandandwalesscotlandandnorthernireland [Accessed 18th June 2021].

[met2061-bib-0030] ONS & Prescott, C. (2020) Internet access—households and individuals. Great Britain: Office for National Statistics. Available from: https://www.ons.gov.uk/peoplepopulationandcommunity/householdcharacteristics/homeinternetandsocialmediausage/bulletins/internetaccesshouseholdsandindividuals/2020 [Accessed 18th June 2021]

[met2061-bib-0031] Popescu, F. & Ionel, I. (2010) Anthropogenic air pollution sources. Air quality, pp. 1–22.

[met2061-bib-0032] Sicard, P. , De Marco, A. , Agathokleous, E. , Feng, Z. , Xu, X. , Paoletti, E. et al. (2020) Amplified ozone pollution in cities during the COVID‐19 lockdown. Science of the Total Environment, 735, 139542. 10.1016/j.scitotenv.2020.139542 32447070 PMC7237366

[met2061-bib-0033] Siddiqui, A. , Halder, S. , Chauhan, P. & Kumar, P. (2020) COVID‐19 pandemic and City‐level nitrogen dioxide (NO_2_) reduction for urban Centres of India. Journal of the Indian Society of Remote Sensing, 48, 999–1006. 10.1007/s12524-020-01130-7

[met2061-bib-0034] Sofia, D. , Gioiella, F. , Lotrecchiano, N. & Giuliano, A. (2020) Mitigation strategies for reducing air pollution. Environmental Science and Pollution Research, 27, 19226–19235. 10.1007/s11356-020-08647-x 32279263

[met2061-bib-0035] StatCounter Global Stats . (2021) Mobile operating system market share United Kingdom. StatCounter Global Stats. Available from: https://gs.statcounter.com/os-market-share/mobile/united-kingdom/#monthly-202101-202106 [Accessed 20th July 2021].

[met2061-bib-0036] Venter, Z.S. , Aunan, K. , Chowdhury, S. & Lelieveld, J. (2020) COVID‐19 lockdowns cause global air pollution declines. Proceedings of the National Academy of Sciences of the USA, 117, 18984–18990. 10.1073/pnas.2006853117 32723816 PMC7430997

[met2061-bib-0037] Voosen, P. (2021) Climate panel confronts implausibly hot models. Science, 373, 474–475. 10.1126/science.373.6554.474 34326213

[met2061-bib-0038] Watts, J. & Kommenda, N. (2020) Coronavirus pandemic leading to huge drop in air pollution. *The Guardian* . Retrieved from: 4th April 2020.

[met2061-bib-0039] World Health Organization . (2020) Statement on the second meeting of the International Health Regulations (2005) Emergency Committee regarding the outbreak of novel coronavirus (2019‐nCoV). Available from: https://www.who.int/news/item/30‐01‐2020‐statement‐on‐the‐second‐meeting‐of‐the‐international‐health‐regulations‐(2005)‐emergency‐committee‐regarding‐the‐outbreak‐of‐novel‐coronavirus‐(2019‐ncov) [Accessed 1st July 2020].

[met2061-bib-0040] Wu, X. , Nethery, R.C. , Sabath, M.B. , Braun, D. & Dominici, F. (2020) Air pollution and COVID‐19 mortality in the United States: strengths and limitations of an ecological regression analysis. Science Advances, 6, eabd4049. 10.1126/sciadv.abd4049 33148655 PMC7673673

[met2061-bib-0041] Wu, Y. , Jing, W. , Liu, J. , Ma, Q. , Yuan, J. , Wang, Y. et al. (2020) Effects of temperature and humidity on the daily new cases and new deaths of COVID‐19 in 166 countries. Science of the Total Environment, 729, 139051. 10.1016/j.scitotenv.2020.139051 32361460 PMC7187824

[met2061-bib-0042] Yechezkel, M. , Weiss, A. , Rejwan, I. , Shahmoon, E. , Ben‐Gal, S. & Yamin, D. (2021) Human mobility and poverty as key drivers of COVID‐19 transmission and control. BMC Public Health, 21, 596. 10.1186/s12889-021-10561-x 33765977 PMC7993906

